# Precision of manual two-dimensional segmentations of lung and liver metastases and its impact on tumour response assessment using RECIST 1.1

**DOI:** 10.1186/s41747-017-0015-4

**Published:** 2017-10-30

**Authors:** F. H. Cornelis, M. Martin, O. Saut, X. Buy, M. Kind, J. Palussiere, T. Colin

**Affiliations:** 1 University Bordeaux, IMB, UMR 5251; CNRS, IMB, UMR 5251; Bordeaux INP, IMB, UMR 5251, Talence, France; 2 INRIA Bordeaux-sud-Ouest, team MONC, 200 Avenue de la Vieille Tour, 33405 Talence, France; 3 Department de Radiologie, Hôpital Tenon, 4 rue de la Chine, 75020 Paris, France; 4 Départment de Radiologie, Institut Bergonié, 229 cours de l’Argonne, 33076 Bordeaux, France

**Keywords:** Computed tomography, Lung, Liver, Metasatses, Response evaluation criteria in solid tumours (RECIST), Segmentation

## Abstract

**Background:**

Response evaluation criteria in solid tumours (RECIST) has significant limitations in terms of variability and reproducibility, which may not be independent. The aim of the study was to evaluate the precision of manual bi-dimensional segmentation of lung, liver metastases, and to quantify the uncertainty in tumour response assessment.

**Methods:**

A total of 520 segmentations of metastases from six livers and seven lungs were independently performed by ten physicians and ten scientists on CT images, reflecting the variability encountered in clinical practice. Operators manually contoured the tumours, firstly independently according to the RECIST and secondly on a preselected slice. Diameters and areas were extracted from the segmentations. Mean standard deviations were used to build regression models and 95% confidence intervals (95% CI) were calculated for each tumour size and for limits of progressive disease (PD) and partial response (PR) derived from RECIST 1.1.

**Results:**

Thirteen aberrant segmentations (2.5%) were observed without significant differences between the physicians and scientists; only the mean area of liver tumours (*p* = 0.034) and mean diameter of lung tumours (*p* = 0.021) differed significantly. No difference was observed between the methods. Inter-observer agreement was excellent (intra-class correlation >0.90) for all variables. In liver, overlaps of the 95% CI with the 95% CI of limits of PD or PR were observed for diameters above 22.7 and 37.9 mm, respectively. An overlap of 95% CIs was systematically observed for area. No overlaps were observed in lung.

**Conclusions:**

Although the experience of readers might not affect the precision of segmentation in lung and liver, the results of manual segmentation performed for tumour response assessment remain uncertain for large liver metastases.

**Electronic supplementary material:**

The online version of this article (doi:10.1186/s41747-017-0015-4) contains supplementary material, which is available to authorized users.

## Key points

- In the lung, uncertainty decreased as diameter of segmented tumour increased
- In the liver, uncertainty increased as diameter of segmented tumour increased
- The accuracy of manual segmentation is not by the experience of the operator

## Background

Tumour progression and response to treatment are currently evaluated according to response criteria based on morphologic imaging such as those firstly proposed by the World Health Organization (WHO) or by the more widely used Response evaluation criteria in solid tumours (RECIST) [1, 2]. Developed to simplify the assessment of tumour response, these two evaluation systems are based on the measurement of a given tumour along the greatest axes, corresponding to an assessment of anatomical tumour burden and changes in the measure over time, with the ultimate goal of categorizing adequate tumour response [3].

RECIST has been demonstrated to be useful in clinical trials where objective response was the primary study endpoint as well as in trials where assessment of stable disease, tumour progression or time-to-progression analyses were undertaken [2, 4]. In RECIST, measurable disease is defined by the presence of at least one measurable lesion [5]. Target tumours should be selected on the basis of their size and be representative of all involved organs, but in addition should be those that lend themselves to reproducible repeated measurements. Thus, it is possible to define a partial response (PR), corresponding to at least a 30% decrease in the sum of diameters of target tumours, taking as reference the baseline sum diameters [2]. Progressive disease (PD) is an increase of at least 20% in the sum of diameters of target tumours, taking as reference the smallest sum.

However, RECIST has significant limitations in terms of variability and reproducibility, which may not be independent [6–9]. In practice, the maximal size mensuration or segmentation (in two or three dimensions) are performed manually and concerns remain about the accuracy of such segmentation as a result of interobserver and intraobserver variability [10]. A recent meta-analysis has shown that interobserver relative measurement difference in measuring single tumour burden and calculating the interval change may exceed the 20% cut-off for progression [11]. However, variability decreased when tumour burden was measured by a single observer or assessed by the sum of multiple tumours [11].

The aim of our study was: firstly to evaluate the precision of manual two-dimensional (2D) segmentations depending on organ, reader experience, and segmentation method; and secondly to quantify the uncertainty in tumour response assessment (PR, PD or stable disease) depending on the segmentation precision.

## Methods

This retrospective study was approved by the institutional research ethics board. The requirement for patient informed consent was waived. The authors had full control of the data and the information submitted for publication.

### Study cohort

Data were extracted from our large departmental electronic database of de-identified computed tomography (CT) images involving two university hospitals. Contrast-enhanced CT scans were obtained in the period from 2010 to 2015 using 0.7–1.2 mm pixel spacing, 1.25–5 mm slice thickness, 120 kVp, and different convolution kernels or constructors (General Electrics, Milwaukee, USA; Siemens, Erlangen, Germany; Phillips, Best, Netherlands).

Two investigators selected the tumours to reflect the variability in location, size, and shape of liver and lung metastases, CT acquisition, reconstruction, and body mass, which all affect the contrast-to-noise ratio and therefore the ease of determination of tumour borders. However, tumours were selected irrespective of primary tumour type or other patient demographics. The number of segmentations was calculated to evaluate the precision of manual segmentation depending on reader experience, on different organs, and using two different segmentation methods. The number of tumours and readers involved in this study was adjusted to ensure sufficient statistical power and a total number of image segmentations greater than 500.

#### Image analysis

Datasets were imported into OsiriX, version 5.9 (OsiriX, Geneva, Switzerland), an open source DICOM image analysis suite and picture archiving and communication system workstation designed for the Apple Macintosh platform. Twenty readers independently analysed CT data from 13 identified non-treated index liver and lung metastases (six livers and seven lungs) using two different methods. Ten readers were radiologists with experience ranging from 1 to 25 years (group 1) and ten readers were scientists with basic knowledge on image segmentation (group 2).

Method 1 consisted of selecting the slice for a given tumour where a mensuration of diameter could be performed according to RECIST or WHO methods and subsequent manual contouring of the tumour on this slice in 2D. While not representative of typical radiologic practice, maximal diameter was automatically extracted from this contour in order to simplify the experimental design. Moreover, for patients with multiple tumours, an approximate tumour location was given by a range of slices where the tumour could be located.

Method 2 consisted of performing the same manual contouring, but the readers were aware of the slice number and tumour location. Method 2 was performed after method 1. Both groups performed both methods. Regions of interest (ROIs) were exported to the Federative Platform for Research in Computer Science and Mathematics (PlaFRIM). The PlaFRIM experimental test bed was used to perform the statistical analysis.

#### Statistical analysis

Only adequate segmentations were selected for subsequent evaluation. Segmentations were considered as inadequate if performed at least two slices away from the slice most often selected by all the readers or not only on the pre-identified nodule; these segmentations were excluded from the analysis. A χ^2^ test was used for independence. Mean, minimum/maximum values, and standard deviation (SD) of the tumour diameter and area were obtained according to organ, group of readers, and methods. To minimize the effect of tumour size factor, measurement variability was expressed as a percentage of the mean diameter/area measurement. Thus, mean SD was divided by the mean diameter or area (mean SD/diameter or area). Mean values were compared using Wilcoxon signed rank test.

To determine interobserver agreement, the between-subject SD and within-subject SD of each variable were compared. Intraclass correlation coefficients (ICCs) were calculated based on repeated measures ANOVA [12, 13]. ICC results were interpreted according to the following criteria: poor (ICC <0.50), moderate (0.50 < ICC < 0.75), good (0.75 < ICC < 0.90), and excellent (ICC > 0.90).

The SD was considered to reflect the variation of segmentation. The mean SD of each diameter or area was plotted according to the respective diameter or area of the tumours in lung and liver. A regression analysis was performed to derive the 95% confidence interval (95% CI) of diameter and area in each organ and for each size. This 95% CI reflects the uncertainty of segmentation whatever the diameter or the area of the tumour. The same 95% CI was also applied for the limits of RECIST 1.1 criteria of PD (+20%) and PR (−30%) either on diameter or on area. The purpose was to detect overlap between the 95% CI of diameter or area and limits of PD or PR. The RECIST was extended to area (A) by adapting the limits of PD and PR using the formula A = π r^2^. A cut-off value of diameter or area was determined if identified at the intersection of the overlap. A *p* value greater than 0.050 was considered to indicate a significant difference. All analyses were conducted using Stata 12.0 (StataCorp, College Station, Texas, United States).

## Results

**Fig. 1:**
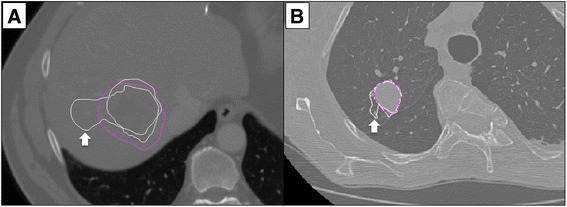
After preselection of the tumours, manual segmentations were performed independently by the operators according to the RECIST and then on a preselected slice. Aberrant segmentations were excluded from the analysis. **a**. Example segmentations performed in liver. The *purple line* corresponds to a segmentation performed by a physician, the *inner line* one performed by a scientist. The outer segmentation (*arrow*) was excluded. **b** Example segmentations performed in lung. The *purple line* corresponds to a segmentation performed by a physician, the *inner line* one performed by a scientist. The outer segmentation (*arrow*) was excluded

A total of 507 segmentations were selected for further evaluation. A total of 13 contours (2.5%, 13/520) were removed due to consistent errors of segmentation, all observed after method 1 (260 segmentations) (Fig. 1). Among these 13 aberrant segmentations, four were performed by radiologists (three in liver, one in lung) and nine by scientists (three liver, six lung). No significant differences were observed between the two groups (*p* = 0.261).

### Interobserver agreement

Between the groups, the mean values did not differ significantly whatever the organ or the method used except only for the mean area of liver tumours (*p* = 0.034) and mean diameter of lung tumours (*p* = 0.021) (Additional file 1). Comparing measurements obtained according to methods 1 and 2, no significant differences were observed between the groups or after combining the groups.

Interobserver agreements were excellent (ICC > 0.90) for all variables; after combining all readings, ICC were 99.1 and 99.4% for diameter and area, respectively.

### Impact on the evaluation of area and maximum diameter

**Fig. 2:**
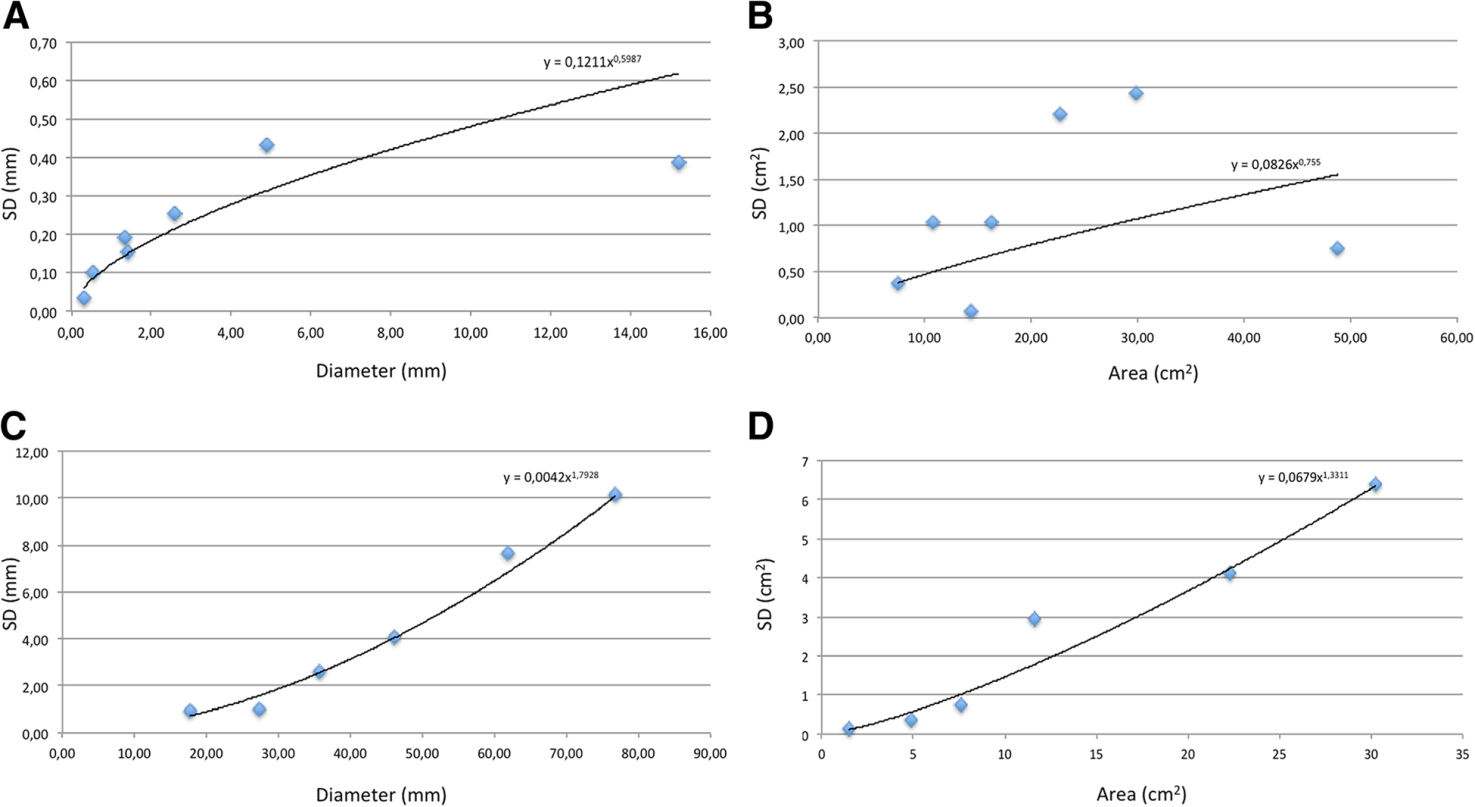
Regression models of the standard deviation according to maximum diameter (in mm) or area (in cm^2^). **a** Mean standard deviation according to the mean maximum diameter of the seven segmentations performed in the lung. A relative dispersion of the mean standard deviation was observed for the maximum diameter but remained below 2.5 mm whatever the size of the segmented tumour. The relative uncertainty decreased with the size. **b** Mean standard deviation according to mean area for each tumour in the lung. **c** Mean standard deviation according to mean maximum diameter for each segmented tumour in the liver (n = 6). The relative uncertainty increased with the size of the lesion. **d** Mean standard deviation according to mean area for each tumour in the liver

**Fig. 3:**
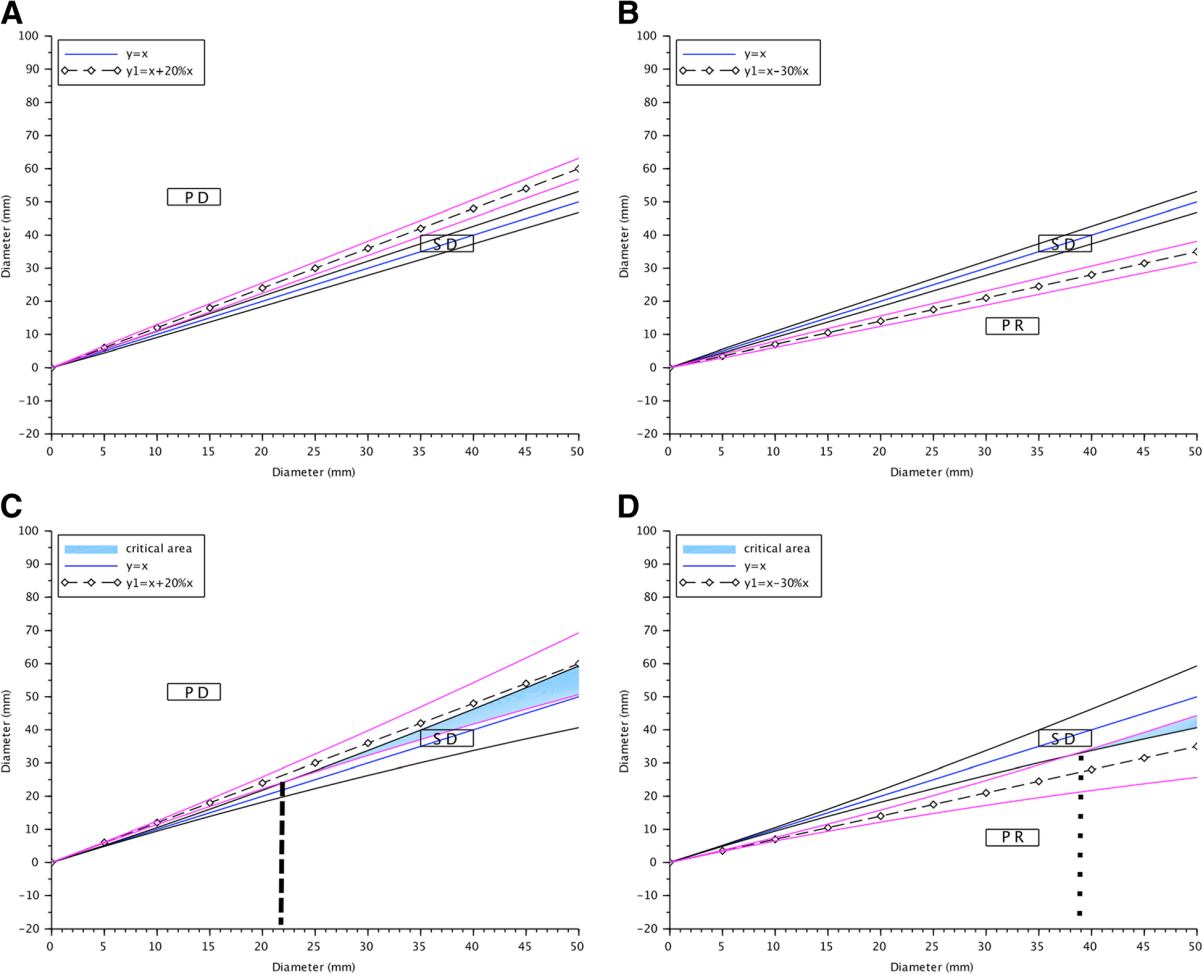
The 95% confidence intervals (95% CIs) obtained for the limits of RECIST 1.1 criteria of stable disease, progressive disease (*PD*), and partial response (*PR*) using diameter. In the lung, it appeared that standard deviation decreased as diameter or area of the segmented tumour increased. The opposite was observed in the liver. **a** The 95% CI of the stable disease (y = x) in the lung did not cross the calculated 95% CI of the lower bound of PD (y = 1.2x). **b** The 95% CI of the stable disease in the lung did not cross the calculated 95% CI of the upper bound of PR (y = 0.7x). **c** The 95% CI of the stable disease in liver shows an overlap (*blue zone*) with 95% CI of the lower bound of PD. The cut-off value was x_1_ = 22.7 mm (*dashed line*). **d** The 95% CI of the stable disease in the liver did cross the calculated 95% CI of the upper bound of PR (*blue zone*). The cut-off value was x_2_ = 37.9 mm (*dashed line*)

**Fig. 4:**
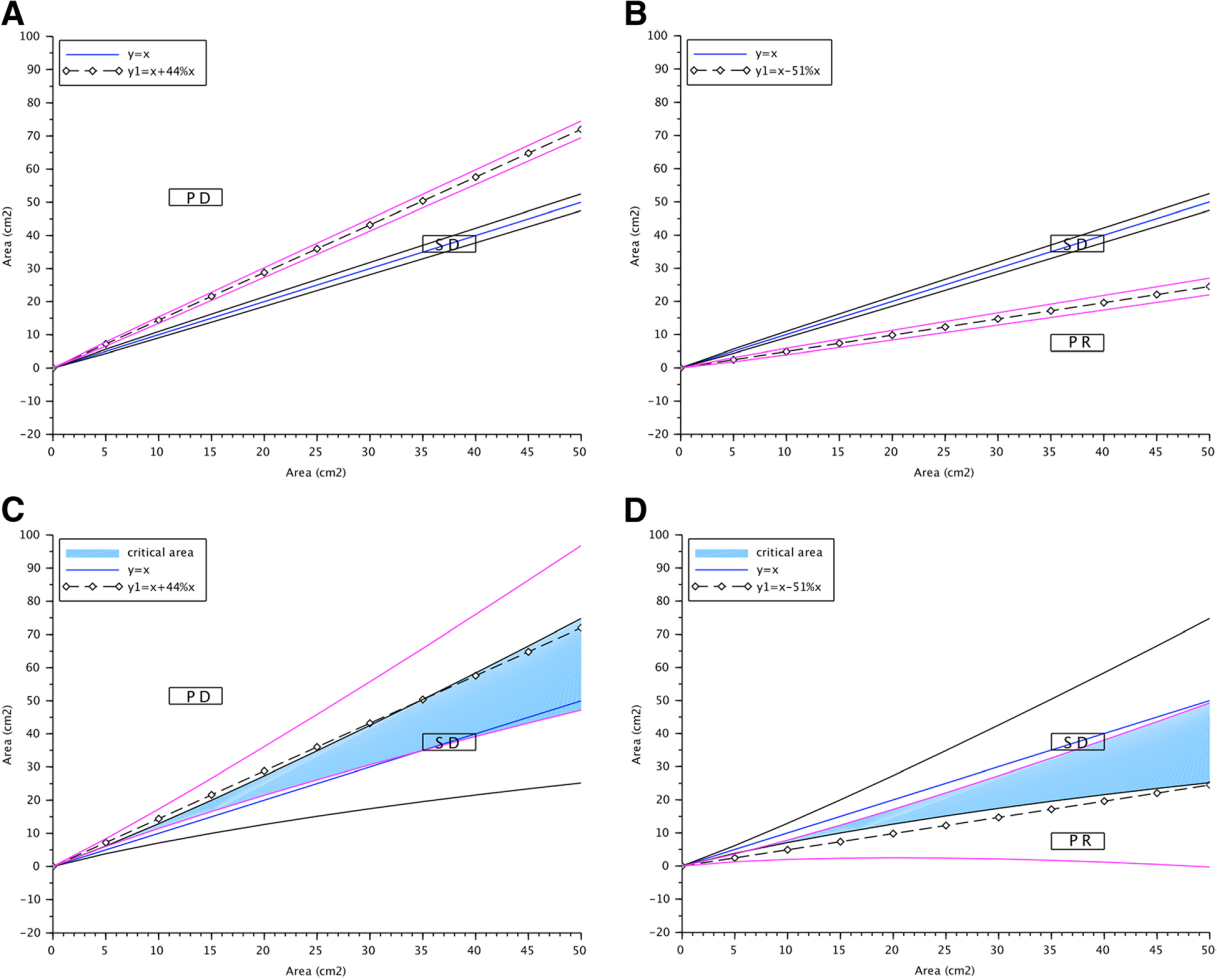
The 95% confidence intervals (95% CIs) obtained for the limits of criteria of stable disease, progressive disease (*PD*), and partial response (*PR*) using area. In liver, the 95% CI of area systematically overlapped across all tumour sizes for both partial response and progressive disease. **a** The 95% CI of the stable disease (y = x) in the lung did not cross the calculated 95% CI of the lower bound of PD (y = 1.44 x). **b** The 95% CI of the stable disease in the lung did not cross the calculated 95% CI of the upper bound of PR (y = 0.47 x). **c** The 95% CI of the stable disease in liver systematically shows an overlap (*blue zone*) with the 95% CI of the lower bound of PD. **d** The 95% CI of the stable disease in the liver always crossed the calculated 95% CI of the upper bound of PR (*blue zone*)

After combination of the values of both groups and both methods (Table 1), regression models were obtained (Fig. 2). After implementation of these regression models in both organs, 95% CIs were successfully calculated for each tumour size and for limits of PD and PR. No overlap of 95% CIs was observed in the lung (Figs. 3a, b and 4a, b). In the liver, the 95% CIs of tumour diameter and area overlapped with the 95% CIs of limits of PD and PR (Figs. 3 and 4). The cut-off value was x_1_ = 22.7 mm at the intersection of the 95% CIs of tumour diameter and limits of PD (Fig. 3c). Similarly, the cut-off was x_2_ = 37.9 mm at the intersection of the 95% CIs of tumour diameter and limits of PR (Fig. 3d). An overlap of 95% CIs was systematically observed for area in liver.

**Table 1:** Overall results of area (cm^2^) or maximum diameter (mm) evaluation for lung and liver lesions

	Area (cm^2^)					Diameter (mm)				
	Mean value	SD	SD/mean	Min	Max	Mean value	SD	SD/mean	Min	Max
Liver 1	1.46	0.14	0.1	1.03	1.61	17.66	0.94	0.05	15.23	19.02
Liver 2	11.66	2.96	0.25	8.46	18.62	46.02	4.06	0.09	40.28	57.06
Liver 3	30.21	6.40	0.21	15.47	45.51	76.72	10.14	0.13	51.83	105.49
Liver 4	4.88	0.37	0.08	3.92	5.78	27.22	1.03	0.04	24.68	29.21
Liver 5	7.58	0.75	0.1	6.39	9.20	35.71	2.61	0.07	31.79	41.74
Liver 6	22.32	4.11	0.18	14.55	27.01	61.77	7.67	0.12	50.97	77.78
Lung 1	1.32	0.19	0.14	0.89	1.60	14.30	0.07	0	12.23	15.83
Lung 2	0.31	0.04	0.13	0.21	0.36	7.43	0.37	0.05	6.51	8.14
Lung 3	0.55	0.10	0.18	0.34	0.77	10.76	1.04	0.1	8.88	12.61
Lung 4	4.87	0.43	0.09	4.06	5.75	29.86	2.43	0.08	24.95	34.52
Lung 5	15.19	0.39	0.03	14.32	15.93	48.78	0.76	0.02	47.33	50.27
Lung 6	2.60	0.26	0.1	1.99	2.96	22.67	2.22	0.1	17.54	27.16
Lung 7	1.42	0.15	0.11	1.18	1.69	16.28	1.03	0.06	14.81	18.33

## Discussion

Among the 520 segmentations performed, only 2.5% of segmented ROIs were removed due to consistent errors of segmentation. No significant difference between radiologists and scientists was observed. Moreover, while considerable interobserver and intraobserver variability has been reported thus far for radiological tumour response evaluation according to RECIST and WHO criteria [11, 14], inter-observer agreements were excellent (ICC >0.90) for diameter and area assessment in both organs.

These observations may be related to the method used in this study. While not representative of typical radiologic practice, the maximum diameter was calculated from an evaluation of the perimeter of the tumour. This analysis was performed to simplify the experimental design and limit the bias. Moreover, for patients with multiple tumours, an approximate tumour location was given by a range of slices where the tumour could be located. While this method appeared effective and confirmed the call for computer-aided detection software for tumour response assessment [15], it remains uncertain how the results are generalizable in clinical practice. Further evaluations are now mandatory.

Based on the regression models of SD performed in this study, the level of uncertainty increased with tumour size in the liver while it decreased in the lung. In liver, therefore, 2D segmentation findings have to be carefully interpreted due to these increasing 95% CIs. A potential impact on tumour response assessment may be observed either for area or for diameter. For area, 95% CIs systematically overlapped. This finding suggests the limited interest of area calculation for therapeutic assessment. For diameter, cut-off values were identified at the intersection of these overlaps, above which it may be difficult to assess confidently therapeutic response. For tumours above these thresholds the impression of progression or partial response may only be related to the uncertainty of the measures. These size limits have to be taken into account in further evaluations of RECIST [2, 16]. This justifies the development of alternatives for liver, such as the recently proposed modified RECIST (mRECIST) [7] or the introduction of functional imaging in the current evaluation of liver metastases after treatment [17, 18]. In the lung, greater uncertainty was observed for small tumours. This finding is consistent with the introduction of a minimum lesion size in RECIST of 10 mm in the lung, which improved reproducibility between WHO and RECIST [19].

This study showed that there were no significant differences in terms of uncertainty between segmentations made by a group of radiologists aware of RECIST and those of a group of scientists with only basic knowledge of RECIST. The SD remained similar for both groups. Moreover, no significant differences were observed between the groups, or after combining the groups, when comparing mensuration obtained on an imposed slice or after the selection of the slice. Therefore, 2D segmentation, even manual, seems not to be affected by a slight variation in slice selection. These findings justify the RECIST 1.1 recommendations to perform mensuration using the same plane of evaluation with the maximum diameter of each target lesion always being measured at subsequent follow-up time points, even if this results in measuring the lesion at a different slice level or in a different orientation or vector compared with the baseline study [2, 16].

This study has some limitations. The series is retrospective and may have selection bias. The number of tumours evaluated is limited and tumours were chosen by two independent investigators, which may have caused selection bias. No comparison of the findings was performed with the results of a single observer or after summing multiple tumours [11]. The segmentation was performed manually but the diameters were extracted automatically. Further studies may compare the results of manual versus automatic segmentations [20, 21]. Volumetric assessment of the entire tumour has not been performed, as recently proposed [22]. However, volumetric assessment and RECIST have been shown not to be interchangeable, neither technique demonstrating clinical superiority [23, 24].

To summarize, the results of our study highlight the concerns remaining for manual segmentation, although accuracy of manual 2D segmentation does not appear to be limited by the experience of operator. For liver but not for lung metastases, segmentation in 2D for response assessment remains uncertain for large tumours. We established thresholds above which the impression of tumour progression or response may be related only to the uncertainty of 2D segmentation. While a prospective validation of these findings on a larger scale is now needed before drawing definitive conclusions regarding their true impact from a clinical perspective, these results could be easily incorporated in daily clinical practice. Moreover, it may justify the development of alternative quantitative assessment of tumour response using multiparametric or functional imaging tools.

## Additional file

Additional file 1: Table S1. Summary of the results for each group in the liver and the lung according to the two methods. (DOC 75 kb)
