# Supplementary material for: Precision of manual two-dimensional segmentations of lung and liver metastases and its impact on tumour response assessment using RECIST 1.1
Source: Eur Radiol Exp. 2017 Oct 30;1:16. doi: 10.1186/s41747-017-0015-4 (PMC5909353; doi:10.1186/s41747-017-0015-4)
Supplement: Supplementary file 1 — Summary of the results for each group in the liver and the lung according to the two methods. (DOC 75 kb) [file 41747_2017_15_MOESM1_ESM.doc]

**Additional file 1**

**Table S1**: Summary of the results for each group in the liver and the lung according to the 2 methods

|  | | | **Method 1** | | | | | | **Method 2** | | | | | |
| --- | --- | --- | --- | --- | --- | --- | --- | --- | --- | --- | --- | --- | --- | --- |
|  |  |  | **Physicians** | | | **Scientists** | | | **Physicians** | | | **Scientists** | | |
|  |  |  | **Mean** | **Std Dev** | **Std Dev/**  **Mean** | **Mean** | **Std Dev** | **Std Dev/**  **Mean** | **Mean** | **Std Dev** | **Std Dev/**  **Mean** | **Mean** | **Std Dev** | **Std Dev/**  **Mean** |
| **Liver** | **Diameter**  **(mm)** | **1** | 17,63 | 0,75 | 0,04 | 17,68 | 1,14 | 0,06 | 17,82 | 0,53 | 0,03 | 18,83 | 2,34 | 0,12 |
|  |  | **2** | 48,55 | 4,08 | 0,08 | 43,49 | 1,98 | 0,05 | 49,36 | 4,23 | 0,09 | 44,58 | 1,79 | 0,04 |
|  |  | **3** | 79,14 | 13,34 | 0,17 | 74,31 | 5,14 | 0,07 | 77,22 | 10,76 | 0,14 | 74,36 | 4,58 | 0,06 |
|  |  | **4** | 27,45 | 0,90 | 0,03 | 27,00 | 1,14 | 0,04 | 28,19 | 1,48 | 0,05 | 27,50 | 1,48 | 0,05 |
|  |  | **5** | 35,52 | 2,33 | 0,07 | 35,92 | 3,02 | 0,08 | 36,14 | 1,49 | 0,04 | 36,82 | 3,44 | 0,09 |
|  |  | **6** | 65,96 | 3,02 | 0,05 | 57,59 | 8,77 | 0,15 | 65,03 | 5,10 | 0,08 | 58,60 | 6,72 | 0,11 |
|  | **Area**  **(cm^2^)** | **1** | 1,45 | 0,10 | 0,07 | 1,47 | 0,18 | 0,12 | 1,48 | 0,16 | 0,11 | 1,57 | 0,29 | 0,18 |
|  |  | **2** | 13,48 | 3,23 | 0,24 | 9,84 | 0,83 | 0,08 | 13,47 | 2,99 | 0,22 | 10,17 | 0,78 | 0,08 |
|  |  | **3** | 32,81 | 8,04 | 0,25 | 27,88 | 3,41 | 0,12 | 32,77 | 7,87 | 0,24 | 28,59 | 3,41 | 0,12 |
|  |  | **4** | 5,01 | 0,34 | 0,07 | 4,75 | 0,37 | 0,08 | 4,99 | 0,49 | 0,10 | 4,79 | 0,45 | 0,09 |
|  |  | **5** | 7,60 | 0,63 | 0,08 | 7,55 | 0,91 | 0,12 | 7,79 | 0,62 | 0,08 | 7,92 | 1,20 | 0,15 |
|  |  | **6** | 25,55 | 1,54 | 0,06 | 19,09 | 3,15 | 0,17 | 24,96 | 2,77 | 0,11 | 21,17 | 4,18 | 0,20 |
| **Lung** | **Diameter**  **(mm)** | **1** | 14,51 | 0,68 | 0,05 | 14,08 | 1,22 | 0,09 | 14,96 | 0,60 | 0,04 | 14,08 | 1,13 | 0,08 |
|  |  | **2** | 7,32 | 0,22 | 0,03 | 7,58 | 0,50 | 0,07 | 7,47 | 0,47 | 0,06 | 7,62 | 1,00 | 0,13 |
|  |  | **3** | 10,52 | 1,13 | 0,11 | 11,04 | 0,91 | 0,08 | 10,89 | 0,58 | 0,05 | 11,08 | 0,83 | 0,07 |
|  |  | **4** | 29,86 | 1,90 | 0,06 | 29,86 | 3,04 | 0,10 | 29,22 | 1,43 | 0,05 | 29,70 | 2,40 | 0,08 |
|  |  | **5** | 48,63 | 0,75 | 0,02 | 48,93 | 0,77 | 0,02 | 49,27 | 0,66 | 0,01 | 49,01 | 0,57 | 0,01 |
|  |  | **6** | 22,32 | 1,57 | 0,07 | 23,02 | 2,77 | 0,12 | 22,67 | 1,02 | 0,05 | 22,95 | 0,90 | 0,04 |
|  |  | **7** | 15,90 | 0,79 | 0,05 | 16,65 | 1,16 | 0,07 | 16,82 | 1,18 | 0,07 | 17,26 | 1,65 | 0,10 |
|  | **Area**  **(cm^2^)** | **1** | 1,37 | 0,11 | 0,08 | 1,28 | 0,25 | 0,20 | 1,43 | 0,09 | 0,06 | 1,29 | 0,24 | 0,18 |
|  |  | **2** | 0,30 | 0,02 | 0,07 | 0,32 | 0,05 | 0,15 | 0,33 | 0,02 | 0,05 | 0,34 | 0,07 | 0,22 |
|  |  | **3** | 0,51 | 0,08 | 0,15 | 0,60 | 0,11 | 0,18 | 0,55 | 0,05 | 0,09 | 0,60 | 0,10 | 0,17 |
|  |  | **4** | 4,87 | 0,40 | 0,08 | 4,87 | 0,49 | 0,10 | 4,69 | 0,12 | 0,03 | 4,68 | 0,30 | 0,06 |
|  |  | **5** | 15,01 | 0,21 | 0,01 | 15,37 | 0,44 | 0,03 | 15,10 | 0,23 | 0,02 | 15,18 | 0,43 | 0,03 |
|  |  | **6** | 2,54 | 0,20 | 0,08 | 2,65 | 0,30 | 0,11 | 2,54 | 0,20 | 0,08 | 2,43 | 0,24 | 0,10 |
|  |  | **7** | 1,34 | 0,11 | 0,08 | 1,50 | 0,15 | 0,10 | 1,44 | 0,12 | 0,08 | 1,51 | 0,20 | 0,13 |

**Note: Std Dev= standard deviation. Method 1 consisted in selecting the computed tomography slice for a given tumour where a mensuration of diameter could be performed according to RECIST or WHO methods and to subsequently contour manually the tumour on this slice in 2D. Method 2 consisted in performing the same manual contour, but the readers were aware of the slice's number and tumour location.**
